# The Polymorphism in Various Milk Protein Genes in Polish Holstein-Friesian Dairy Cattle

**DOI:** 10.3390/ani11020389

**Published:** 2021-02-03

**Authors:** Magdalena Kolenda, Beata Sitkowska

**Affiliations:** Department of Animal Biotechnology and Genetics, Faculty of Animal Breeding and Biology, UTP University of Science and Technology, 85-084 Bydgoszcz, Poland; beatas@utp.edu.pl

**Keywords:** casein, PAEP, β-lactoglobulin, frequency, microarrays

## Abstract

**Simple Summary:**

The aim of the present study was to evaluate the genetic variability within casein alpha S1 (CSN1S1), casein alpha S2 (CSN1S2), beta-casein (CSN2), kappa-casein (CSN3), and progestagen-associated endometrial protein (PAEP) genes. The study was conducted on 1900 Polish Black and White Holstein-Friesian dairy cows that were genotyped using customized EuroGenomics microarrays. Out of investigated 24 genetic variants within four genes coding milk protein, 16 expressed genetic diversity. The fact that many of the investigated Single Nucleotide Polymorphisms (SNPs) were monomorphic may suggest that in the past the reproduction program favored one of these genotypes. Populations of high-yielding cows become more and more even and similar in terms of genotype, which may have an adverse effect in future breeding, therefore, the variability in genetic variants of genes coding milk protein should be monitored.

**Abstract:**

The aim of the present study was to evaluate the genotype and allele frequencies of 24 polymorphisms in casein alpha S1 (CSN1S1), casein alpha S2 (CSN1S2), beta-casein (CSN2), kappa-casein (CSN3), and progestagen-associated endometrial protein (PAEP) genes. The study included 1900 Polish Black and White Holstein-Friesian dairy cows that were subjected to genotyping via microarrays. A total of 24 SNPs (Single Nucleotide Polymorphisms) within tested genes were investigated. Two CSN1S1 SNPs were monomorphic, while allele CSN1S1_3*G in CSN1S1_3 SNP dominated with a frequency of 99.39%. Out of seven CSN2 SNPs, four were polymorphic; however, only for CSN2_3 all three genotypes were detected. Only three out of nine SNPs within CSN3 were monomorphic. Three PAEP SNPs were also found to be polymorphic with heterozygotes being most frequent. Hardy–Weinberg equilibrium (HWE) was observed for eight variants. It was shown that only CSN3_6 was not in HWE. The fact that many of investigated SNPs were monomorphic may suggest that in the past the reproduction program favored one of these genotypes. SNPs that are included in commercially available microarrays should be monitored in relation to changes in their frequencies. If a SNP has turned monomorphic, maybe it should be considered for removal from the microarray.

## 1. Introduction

All over the world dairy products made from cow’s milk are a crucial part of human diet. The demand for milk products steadily increases, especially due to the fact that milk is a known source of micro and macronutrients, such as lipids, proteins, vitamins, and minerals [1]. 

The composition of cow’s milk is well known, with 80% being water, 3% to 4% fat, 3.5% proteins, approximately 5% lactose, and 1.2% minerals. It is worth mentioning that milk proteins have a high biological value as they contain several essential amino acids. Caseins make up approximately 80% of all milk proteins in cow’s milk [1]. Caseins may be divided into four forms—casein alpha S1, casein alpha S2, beta-casein, and kappa-casein—and are coded by CSN1S1, CSN1S2, CSN2, and CSN3 genes, respectively. All these genes are located on bovine chromosome 6 [2]. In dairy cattle, nine alleles (A-I) are described for CSN1S1 [3]. The literature describes 12 CSN2 alleles (A1, A2, A3, B, C, D, E, F, H1, H2, I, and G) that can be detected in dairy cattle; however, not all of them are present in all breeds [2]. Different SNPs contribute to the creation of described CSN2 (e.g., A1-A3, B-G, I) and CSN3 (A-E, G1-I) variants [3,4,5,6,7]. 

Apart from caseins, cow’s milk is composed in 20% of whey proteins, one of which is β-lactoglobulin (βLG) coded by the PAEP gene (previously called LBG gene). PAEP is localized on bovine chromosome 11 [3]. Literature describes many genetic variants of that gene, including A, B, and D presented in the Tables S1–S5 [3,4,5,6,7]. Tables included in the Supplementary Materials present genes variants in relation to alleles discussed in this paper.

Currently, the genomic assessment of cattle breeding value that is carried out in many countries, preceded by genotyping of animals with the use of Illumina microarrays, allows obtaining additional information on the variability of SNP located in genes, e.g., determining milk protein variants. Studying the results coming from microarrays allows tracking changes in SNP frequencies in a given population [8] as well as for the further analysis of the relationship between selected polymorphism and the level of dairy cows’ characteristics (e.g., the level of bioactive milk components). Variant frequencies may differ according to breed and even herd. This may suggest that the variability of different milk protein SNPs should be monitored in dairy cattle herds [7,8]. Through the years, a selection aiming at improving milk performance might have changed genotype frequencies of different SNPs, rendering some of them monomorphic. Chessa et al. [8] reported that selection favored one of the alleles for CSN2. They pointed that the direct selection aiming at improving production is shifting genetic variants frequencies, increasing the frequency of A2 and decreasing A1 variant. We hypothesise that the tested populations of cows may be monomorphic in terms of some SNPs. If a SNP is no longer present in different variants, maybe it should be considered for removal from the microarray and replaced by a new SNP that would be of value to breeders. 

The aim of the present study was to evaluate the genotype and allele frequencies of 24 single nucleotide polymorphisms in casein alpha S1, casein alpha S2, beta-casein, kappa-casein, and progestagen-associated endometrial protein genes in the population of Polish Black and White Holstein-Friesian dairy cows. 

## 2. Materials and Methods

In accordance with Resolution No. 13/2016 of the National Ethics Committee for Animal Experiments (Poland) of 17 June 2016, the consent of the Ethics Committee is not required for the collection of animal material for genotyping. 

The study was carried out on a total of 1900 Polish Black and White Holstein-Friesian dairy cows that were born between 2014 and 2016 and calved between 2016 and 2018. Animals were used in a conventional milking system. All studied cows came from 3 high-yielding herds, with the average milk yield per lactation at the level of 12,321.29 kg, mean fat, protein, lactose, and solids contents at the level of 3.65%, 3.34%, 4.87%, and 12.69%, respectively. The genetic material for the study was collected during a routine estimation of breeding value. The genotyping using microarrays was outsourced to the Polish Federation of Cattle Breeders and Dairy Farmers (PFCB&DF, Warszawa, Poland). As a part of Euro Genomics, cooperative PFCB&DF uses customized EuroGenomics arrays. For the purpose of the present study, the following arrays were used: Eurogenomics v3_POL; Eurogenomics v4_POL; Eurogenomics v5_POL; Eurogenomics v6_POL; Eurogenomics v8b_POL; Eurogenomics MD_POL with Infinium HD Illumina protocol. The different panels had very little overlap and due to the limited number of genotypes available, the imputation was not successful, thus, only 6765 genome wide SNPs with a call rate of >0.95 across all animals and minor allele frequency MAF > 0.001 were used. Biopsy samples (ear punch) were collected with the use of AllFlex Tissue Sampling Unit (TSU, Alleflex, Cape Town, South Africa) in the period from 2015 to 2019. Samples were kept in room temperatures and imminently transferred to the laboratory by postal services (2–3 days). In the laboratory, samples were coded and frozen at −20 °C degrees. After unfreezing, samples were transferred to 96 well plates, after which the lysis buffer and proteinase K were added. This mix was then incubated at 56 °C and mixed (600 RPM) in a thermomixer overnight. Before the next step, samples were centrifuged (60 s × 3200 g) to remove undigested parts. Next, lysate was transferred to deep-well plates and DNA extraction was processed with the use of Thermo KingFisher™ Cell and Tissue DNA Kit (Thermo Fisher, Waltham, MA, USA) according to the manufacturer’s protocol in the KingFishre Duo DNA system (Thermo Fisher, Waltham, MA, USA). 

DNA concentration was measured according to Illumina protocol (Infinium HD) by the fluorimetric method with the use of the Quant-iT PicoGreen™ dsDNA Assay Kit (Thermo Fisher, Waltham, MA, USA) in the Fluoroscan™ Microplate Fluorometer (Thermo Scientific, Waltham, MA, USA) system. Samples were then normalized to 70 ng/ul by dilution with a use of TE buffer (Novazym, Poznań, Poland). Normalized samples were processed according to Illumina HD protocol. Beadchips were immediately scanned on the Illumina iScan system, and scans were analyzed using GenomeStudio Software V2011.1 version 1.9.4 (Illumina, San Diego, CA, USA). All samples from one type of array were clusterized again together before export with the use of cluster file. Only data with call rates over 0.95 were chosen to subsequent statistical analysis. Eurogenomics arrays’ content (probe list and sequences) are confidential with underlying restrictions for publication. For the purpose of the present study, authors have chosen SNPs that were present on all array versions. Farmers have authorized the authors to access a portion of the SNP data.

The genotypic data regarding SNP in milk protein genes was provided by PFCB&DF following farmers’ consent. SNP data for the genes tested in the present study are presented in Table 1, and the comparison to other well-known mutations is presented in the Supplementary Materials (Tables S1–S5). The study focused on three CSN1S1, one CSN1S2, seven CSN2, nine CSN3, and four PAEP SNPs. Allele and genotype frequencies for all SNPs were calculated and the data were subjected to the Hardy–Weinberg equilibrium test (P value for x^2^ test with 1 degree of freedom and α = 0.05).

## 3. Results and Discussion

### 3.1. Allele and Genotype Frequency

It is worth mentioning that in populations with a high number of heterozygous animals in which genetic diversity is still present, it is possible to influence the change in production levels by favouring different alleles while selecting animals for breeding. Since all alleles are present in the population, breeders may design selection programs and select bulls in relation to favorable alleles, so that new and favorable variants would appear in their herds with higher frequency. Therefore, it is necessary to investigate allelic frequencies of different commercially important genes. 

In the present study, 24 SNPs located in milk protein genes were investigated (Table 2). Out of three casein alpha S1 (CSN1S1) genetic variants, only one (CSN1S1_3) was found to be polymorphic, although the frequency of B*B* genotype (c.*31GG) was proven to dominate at the level of 99%. Chessa et al. [8] investigated the polymorphism in CSN1S1 (in the present study marked as CSN1S1_3) in Italian cattle. They reported similar frequency of allele B as in obtained in the present study: in Holstein-Friesian (HF), population frequency was at the level of 0.998, and in Italian Holstein, the population was at 0.997. The frequency of allele B was considerably lower in the Italian Jersey (0.416) population. Polymorphism in CSN1S1 was also described by other authors; however, they reported a different set of SNPs that corresponded to alleles B and C as well as resulting genotypes [9], revealing that genotype BB was the most frequent (90.48%) in the HF breed. Hristov et al. [7] also investigated polymorphism in the CSN1S1 gene, showing B and C alleles at the frequency of 0.621 and 0.379, respectively. 

It is worth noticing that c.222G>T SNP is also included into the makeup of widely recognized CSN1S2 alleles: allele A (contains guanine) and D (thymine). Investigation of SNP within casein alpha-S2 gene (CSN1S2) that resulted in guanine to thymine substitution showed the frequency of c.222TT genotype (which in the literature would be called DD genotype) to be almost at the level of 100%, and the T allele was present in 99.95% of all tested cases (Table 2). Only few animals in the population were heterozygous. Similar results were obtained by Ardicli et al. [9], who also investigated the same SNP and reported that in HF population most frequent was DD genotype (97.62% of all animals). Out of 168 tested animals, only four were heterozygous, having AD genotype. 

Seven SNPs within beta-casein (CSN2) gene were investigated; however, only five were polymorphic. Furthermore, for CSN2_3 (c.350A>C), only three genotypes were detected (A*A*: 14%, A*B*: 47%, B*B*: 38%) (Table 2). Six out of seven investigated SNPs were also included into makeup of well-known CSN2 alleles. The CSN2 SNPs with the highest frequencies (Table 2) correspond to alleles A1 and A2, which suggests that they are most common in the studied population. Other authors also evaluated frequencies in this gene, among others, Chessa et al. [10], who reported five SNP within the CSN2 gene, out of which four were also reported in the present study. They referred to X14711 reference sequence and their SNPs were at the positions 6690, 8101 (according to UMD3.1: CSN2_3, c.245C>A), 8178 (CSN2_4, c.322A>C), 8219 (CSN2_5, c.363C>A), and 8267 (CSN2_6, c.411C>G). CSN2_3 is characterized by the change C>A, and cytosine is present in alleles A2, A3, and I, while adenine in alleles A1, B, and C. In SNP CSN2_4, adenine is present in alleles A1, A2, A3, B, and C, while cytosine in allele I. In case of CSN2_5, cytosine is present in all alleles except for allele A3 where adenine is present. For CSN2_6, guanine is present in allele B, while the rest contain cytosine. Chessa et al. [8] also investigated data coming from microarrays that contained several CSN2 SNPs that were also present in the present study. They reported similar frequencies of allele B* in HF and Italian Holstein populations for CSN2_3 (0.609 and 0.608 compared to 0.619 in the present study), CSN2_5 (0.998 and 0.998 comparing to 0.999), and CSN2_7 (0.997 and 0.999 comparing to 0.999). Higher frequencies of allele B* were detected in SNP that corresponded to CSN2_6 (0.018 and 0.028 in HF and Italian Holstein population, respectively, compared to 0.003 in the present study). The population of Italian Jersey [8] was characterized by higher B* allele frequency for CSN2_3, CSN2_5, and CSN2_7 than in the present study (0.787 vs 0.619; 1 vs 0.999; and 1 vs 0.999, respectively). Sebastiani et al. [2] also studied alleles and genotypes in CSN2 gene, reporting that most frequent were allele A2 (60.65%) and A1 (30.39%). Bonfatti et al. [11], who performed their study on 2167 Simmental cows, also investigated the polymorphism in casein genes, showing frequency for CSN2 A2 allele to be at the level of 0.188, A1 of 0.598, B of 0.158, and I of 0.058. Massella et al. [12], who tested 1230 cows (1226 Holstein-Friesian and 4 Braunvieh cows), reported in the population the occurrence of five variants (A1, A2, B, D, and I) and 13 genotypes. They reported that the most frequent were A2 (0.546) and A1 (0.371) alleles and A1A2 (0.403) and A2A2 (0.301) genotypes. 

Nine SNP in CSN3 were investigated in the present study, and six of them were polymorphic (CSN_3- CSN_6, CSN3_8, CSN3_9). Frequencies of each respective SNP are presented in Table 2. Frequency of allele B* for CSN3_8 was at the level of 0.111, which was at a similar level compared to the frequency obtained by Chessa et al. [8] for that allele in HF (0.159) and Italian Holstein (0.105) cows; however, investigation of that polymorphism in Italian Jersey population revealed a lower frequency of B* allele, suggesting that the frequency is dependent on the breed. Moreover, comparing most frequent SNP variants from the present study with known CSN3 alleles, one may conclude that most frequent were alleles A and B. Bonfatti et al. [11] also confirm the presence of CSN3 genotypes (AA, AB, BB), with AA and AB being present in the population at a similar level (0.438 and 0.432, respectively). Furthermore, Barbosa et al. [13] investigated frequencies of CSN3 gene, reporting that 73% of 867 investigated animals had AA genotype, while 25% were heterozygotes. In the study by Sitkowska et al. [14], carried out on 398 Polish Holstein-Friesian of the Black and White variety (PHF), genotype AA was also most frequent (69%) while BB was the least frequent (7%). Similar results were presented by Sitkowska et al. [15] (304 PHF), with 71% of animals being AA homozygotes and 23% heterozygotes. Adamov et al. [16], studying the polymorphism in CSN3 in randomly selected cows, confirmed the presence of five genotypes (AA, AB, BB, AE, and BE); however, genotypes AA, AB, and BB were most frequent (38.8%, 29.2%, and 16%, respectively), adding up to a total of 84% of all detected genotypes, which might suggest that due to favorable consequences, the presence of these genotypes was increased due to selection. 

The study also evaluated frequencies of four PAEP SNPs, indicating that one was monomorphic (PAEP_1). For other three SNPs, most frequent were heterozygotes (PAEP_2: 0.52%, PAEP_3: 0.51%, PAEP_4: 0.51%) (Table 2). Comparing these results to the well PAEP alleles described in the literature [3,4,5,6], one may note that most frequent were A and B alleles. Other authors also investigated polymorphism in the PAEP gene, one of them being Bonfatti et al. [11], who reported in the tested population the presence of three alleles (A, B, and D) and five genotypes (AA, AB, AD, BB, and BD). They stated that the frequency of A allele was the highest (0.543), followed by B frequency (0.449). They reported that the highest frequency was reported for AB genotype (46.6%), while AA (30.6%) and BB (21.3%) were less frequent. Moreover, Sitkowska et al. [14] and [17] reported that the AB genotype was most frequent (44% and 50.32%, respectively) in the investigated population of PHF. Different results were obtained by Akter et al. [18], who investigated the polymorphism in the PAEP gene in Bangladesh cattle, reporting that the genotype BB was the most frequent (66%), while AA and AB were at a similar level (16% and 18% respectively). They did not, however, provide information on cattle breed, indicating that cows were selected randomly, which may suggest that different breeds might have been subjected to the test. Differences in allele B frequency may suggest that frequency of each allele in the PAEP gene are breed dependent. 

### 3.2. Hardy–Weinberg Equilibrium

The studied population was tested whether it was in Hardy–Weinberg (H-W) equilibrium in relation to investigated SNPs (Table 2). Since the test is not accurate if any of the genotypes has less than five individuals, 17 SNPs were excluded from the test, as at least one of the genotypes did not appear in the population. In terms of CSN1S1 and CSN1S2, the tested population was not in accordance with the Hardy–Weinberg model, since it was monomorphic for three SNPs and near fixation for CSN1S1_3, therefore, Hardy–Weinberg equilibrium χ^2^ was not applicable. This may suggest that in the studied population one of the genotypes was favored during reproduction programs in the past. Other authors also described results of χ^2^ test used to verify any deviation from H-W equilibrium, for instance, Ardicli et al. [9] who investigated polymorphism in CSN1S1, noted that the tested HF population was not in H-W equilibrium (not all genotypes were present in the population, χ^2^ was at the level of 0.42, and *p*-value at 0.52), similarly with CSN1S2 genotypes. Furthermore, Hristov et al. [7] subjected their result to χ^2^ test in order to evaluate the validity of H-W equilibrium. They investigated polymorphism in CSN1S1 in Bulgarian Rhodopean Cattle (BRC) and noted that while χ^2^ (0.26) and *p*-value (0.88) suggest that observed and expected frequencies did not vary significantly and the population was in H-W equilibrium, the relatively low frequency of CC genotypes (0.023) might suggest that in the past animals with BB and/or BC genotypes were preferred and used during reproduction in BRC breed. 

Only for one CSN2 SNP, all three genotypes were detected rendering H-W equilibrium χ^2^ applicable. Observed frequencies for CSN2_3 did not differ from those expected (χ^2^ test *p* value at the level of 0. 0.843, was greater than α = 0.05), which suggests that there was no significant difference from H-W prediction (Table 2). Sebastiani et al. [2] also stated that no deviation from equilibrium was detected in their study. Literature, however, provides a description of studies in which observed and expected heterozygosity significantly differed (*p* < 0.05) confirming that the population was not in H-W equilibrium [19]. Such results may suggest that in the studied population management focusing on reproduction and genetic improvement might have been applied. 

Out of nine CSN3 SNPs, four were tested whether they were in accordance with H-W model, for others the test was not applicable due to the monomorphisms of the population or the lack of presence of all genotypes (Table 2). Out of four SNPs in CSN3 that were subjected to H-W test, three were in Hardy–Weinberg equilibrium (CSN3_5, CSN3_8, CSN3_9) and their observed and expected frequencies did not differ significantly, while SNP CSN3_6 with χ^2^ at the level of 6.69 and *p*-value at 0.01 was out of Hardy–Weinberg equilibrium. Another study that described a population with significantly similar observed and expected genotype frequencies and conformed validity of H-W equilibrium was conducted by Hristov et al. [7]. In the research of Barbosa et al. [13], however, observed frequencies for CSN3 loci differ from Hardy–Weinberg equilibrium (χ^2^ = 0.00, *p* < 0.01), which might suggest that in the past selection during reproduction, that favored one of the genotypes, occurred. 

The study investigated four SNPs in the PAEP gene, out of which one was monomorphic, while three polymorphic SNPs were proven to be in H-W equilibrium (Table 2). Barbosa et al. [13] also reported polymorphism in the PAEP gene (both A and B alleles and resulting genotypes were present in the population). In their study, observed frequencies did not differ significantly from expected ones, suggesting that the population was in Hardy–Weinberg equilibrium (χ^2^ = 1.04; α = 0.01). Similarly, population of BRC cattle investigated by Hristov et al. [7] was in agreement with H-W principles (χ^2^ = 0.13, *p*-value = 0.94). 

It is worth noting that the Hardy–Weinberg model is often used in literature as the base model for evolution. Therefore, if any population is proven to be out of Hardy–Weinberg equilibrium, it may suggest that some kind of evolutionary process may affect the population. Deviation from H-W equilibrium may be also an indicator that matings in the herd were planned favoring one of the genotypes [19].

## 4. Conclusions

Milk proteins are important from an economical point-of-view as the polymorphism between different protein genes and milk and cheese properties have been wildly described. The information on these genotypes is important for breeders when they want to improve their herds. However, years of selection may have caused significant changes in the frequencies of different genotypes. The fact that all investigated SNPs for CSN1S1 and CSN1S2 and many SNPs in CSN2 and CSN3 genes were monomorphic may suggest that in the past the reproduction program favored one of these genotypes. Therefore, it is worth monitoring the frequencies of different SNPs, especially if they are included in commercially available microarrays. If a monomorphic SNP is included in microarray, it generates unnecessary costs in production, and it also takes up a place that a different SNP, also beneficial for farmers, can take. 

## Figures and Tables

**Table 1:** Single Nucleotide Polymorphism (SNP) information (UMD3.1 reference genome).

SNP Name	Chromosome	Position on the Chromosome	Consequences	Reference SNP ID Number	Polymorphism Name	
CSN1S1_1	6	87148464	missense variant	rs433385179	c.202G>A	p.Ala68Thr
CSN1S1_2	6	87150805	missense variant	rs132656458	c.272T>C	p.Val91Ala
CSN1S1_3	6	87157909	3 prime UTR variant	rs133474041	c.*31G>A	.
CSN1S2	6	87268 830	missense variant	rs463985801	c.222G>T	p.Glu74Asp
CSN2_1	6	87181376	missense variant	rs454083280	c.488A>C	p.His163Pro
CSN2_2	6	87183031	missense variant	rs721259074	c.154G>A	p.Glu52Lys
CSN2_3	6	87181619	missense variant	rs43703011	c.245C>A	p.Pro82His
CSN2_4	6	87181542	missense variant	rs109299401	c.322A>C	p.Met108Leu
CSN2_5	6	87181501	missense variant	rs43703012	c.363C>A	p.His121Gln
CSN2_6	6	87181453	missense variant	rs43703013	c.411C>G	p.Ser137Arg
CSN2_7	6	87181364	missense variant	rs433954503	c.500C>T	p.Pro167Leu
CSN3_1	6	87390458	missense variant	.	c.352C>T	p.Arg118Cys
CSN3_2	6	87390459	missense variant	rs716557965	c.353G>A	p.Arg118His
CSN3_3	6	87390479	missense variant	rs43706475	c.373T>G	p.Ser125Ala
CSN3_4	6	87390573	missense variant	rs450402006	c.467C>T	p.Thr156Ile
CSN3_5	6	87390576	missense variant	rs43703015	c.470T>C	p.Ile157Thr
CSN3_6	6	87390612	missense variant	rs43703016	c.506C>A	p.Ala169Asp
CSN3_7	6	87390619	synonymous variant	rs439304887	c.513A>G	p.Pro171=
CSN3_8	6	87390632	missense variant	rs43703017	c.526A>G	Ser176Gly
CSN3_9	6	87390673	synonymous variant	rs110014544	c.567G>A	p.Ala189=
PAEP_1	11	103302553	missense variant	rs211077340	c.181G>C	p.Glu61Gln
PAEP_2	11	103303473	synonymous variant	rs110180463	c.237C>T	p.Asn79=
PAEP_3	11	103304668	synonymous variant	rs110641366	c.312C>T	p.Asn104=
PAEP_4	11	103304757	missense variant	rs109625649	c.401T>C	p.Val134Ala

Abbreviations: synonymous variant, A—Adenine, C—cytosine, G—guanine, T—Thymine, Ala—Alanine, Arg—Arginine, Asn—Asparagine, Asp—Aspartic acid, Cys—Cysteine, Gln—Glutamine, Glu—Glutamic acid, Gly—Glycine, His—Histidine, Ile—Isoleucine, Leu—Leucine, Lys—Lysine, Met—Methionine, Pro—Proline, Ser—Serine, Thr—Threonine, Val—Valine.CSN1S1—casein alpha S1, CSN1S2—casein alpha S2, CSN2—beta-casein, CSN3—kappa-casein, PAEP—progestagen-associated endometrial protein.

**Table 2:** Allele and genotype frequency (%) in tested genes.

SNP Name	Number of Cows (n)	Allele A* (nt)	Allele B* (nt)	Genotype Frequency (%)			Allele Frequency (%)		χ^2^ (HWE)	χ^2^ Test *p*-Value
				A*A*	A*B*	B*B*	A*	B*		
CSN1S1_1	1897	A	G	0.00	0.00	1.00	0.00	100.00	.	.
CSN1S1_2	1900	C	T	1.00	0.00	0.00	100.00	0.00	.	.
CSN1S1_3	1900	A	G	0.00	0.01	0.99	0.61	99.39	.	.
CSN1S2	1900	G	T	0.00	0.00	1.00	0.05	99.95	.	.
CSN2_1	1897	G	T	1.00	0.00	0.00	100.00	0.00	.	.
CSN2_2	1896	T	C	0.00	0.00	1.00	0.00	100.00	.	.
CSN2_3	1896	A	C	0.14	0.47	0.38	38.01	61.99	0.039	0.843
CSN2_4	1893	A	C	0.88	0.12	0.00	93.86	6.14	.	.
CSN2_5	1897	A	C	0.00	0.00	1.00	0.08	99.92	.	.
CSN2_6	1897	C	G	0.93	0.07	0.00	96.68	3.32	.	.
CSN2_7	1885	C	T	0.00	0.00	1.00	0.08	99.92	.	.
CSN3_1	1900	C	T	0.00	0.00	1.00	0.00	100.00	.	.
CSN3_2	1900	A	G	0.00	0.00	1.00	0.00	100.00	.	.
CSN3_3	1869	G	T	0.98	0.02	0.00	99.20	0.80	.	.
CSN3_4	1865	C	T	0.00	0.02	0.98	0.88	99.12	.	.
CSN3_5	1897	C	T	0.16	0.47	0.36	40.04	59.96	0.574	0.449
CSN3_6	1824	A	C	0.38	0.45	0.17	60.64	39.36	6.690	0.010
CSN3_7	1900	A	G	1.00	0.00	0.00	100.00	0.00	.	.
CSN3_8	1894	A	G	0.79	0.20	0.01	88.89	11.11	1.575	0.210
CSN3_9	1892	A	G	0.36	0.47	0.16	60.10	39.90	0.415	0.520
PAEP_1	1900	G	C	1.00	0.00	0.00	100.00	0.00	.	.
PAEP_2	1892	C	T	0.29	0.52	0.19	54.78	45.22	3.054	0.081
PAEP_3	1894	C	T	0.18	0.51	0.31	43.19	56.81	2.049	0.152
PAEP_4	1894	C	T	0.31	0.51	0.18	56.81	43.19	2.049	0.152

A*, B*—allele names used in the present study for SNPs included in the microarray, χ^2^ (HWE)—Hardy–Weinberg equilibrium χ^2^ value; χ^2^ test *p*-value—χ^2^ test *p* value with 1 degree of freedom and α = 0.05; Hardy–Weinberg (HW) equilibrium was calculated only if any genotype group had at least 5 individuals.

## Data Availability

Data is contained within the article or supplementary material.

## Supplementary Materials

The following are available online at https://www.mdpi.com/2076-2615/11/2/389/s1, Table S1: Selected CSN1S1 genetic variants in relation to nucleotide position, amino acid change within various *Bos genus* CSN1S1 alleles described in the literature [3,5,6,7,8], Table S2: Selected CSN1S2 variants in relation to nucleotide position, amino acid change within various *Bos genus* CSN1S2 alleles described in the literature [3,5,6,7,9], Table S3: Selected CSN2 genetic variants in relation to nucleotide position, amino acid change within various *Bos genus* CSN2 alleles described in the literature [3,5,6,7], Table S4: Selected CSN3 genetic variants in relation to nucleotide position, amino acid change within various *Bos genus* CSN3 alleles described in the literature [3,5,6,7,8], Table S5: Selected PAEP genetic variants in relation to nucleotide position, amino acid change within various *Bos genus* PAEP alleles described in the literature [3,5,6,7].
