# Supplementary material for: The Polymorphism in Various Milk Protein Genes in Polish Holstein-Friesian Dairy Cattle
Source: Animals (Basel). 2021 Feb 3;11(2):389. doi: 10.3390/ani11020389 (PMC7913634; doi:10.3390/ani11020389)
Supplement: Supplementary file 1 [file animals-11-00389-s001.pdf]

animals MDPI

The Polymorphism in Various Milk Protein Genes in Polish Holstein-Friesian Dairy Cattle

Magdalena Kolenda* and Beata Sitkowska

Table S1. Selected CSN1S1 genetic variants in relation to nucleotide position, amino acid change within various *Bos genus* CSN1S1 alleles described in the literature [3,5–8].

Nucleotide Position *	Allele (Nucleotide/Amino Acid)						
	A	B	C	D	E	F	I
14891-14929	Del.						
17383		G/Ala		A/Thr			
17807		A/Glu	G/Gly				
18901		C/Gln			A/Lys		
18923		C/Ser				T/Leu	
19836		A/Glu					T/Asp
26181		A/Glu	G/Gly		G/Gly		

* on the basis of the bovine genomic sequence GenBank Accession No. X59856; Abbreviations used in tables 1–7: Del.—deleted, A—Adenine, C—cytosine, G—guanine, T—Thymine; Ala—Alanine, Arg—Arginine, Asn—Asparagine, Asp—Aspartic acid, Cys—Cysteine, Gln—Glutamine, Glu—Glutamic acid, Gly—Glycine, His—Histidine, Ile—Isoleucine, Leu—Leucine, Lys—Lysine, Met—Methionine, Pro—Proline, Ser—Serine, Thr—Threonine, Val—Valine.

Table S2. Selected CSN1S2 variants in relation to nucleotide position, amino acid change within various *Bos genus* CSN1S2 alleles described in the literature [3,5–7,9].

Nucleotide Position *	SNP Name **	Allele (Nucleotide/Amino Acid)			
		A	B	C	D
6227		C/Ser	T/Phe		
7568		A/Glu		G/Gly	
8401		G/Ala		A/Thr	
8879	CSN1S2	G/Glu			T/(Asp)-del
11018		C/Thr		T/Ile	

*on the basis of the bovine genomic sequence GenBank Accession No. M94327; ** the name assigned to SNP that was present in microarrays.

Table S3. Selected CSN2 genetic variants in relation to nucleotide position, amino acid change within various *Bos genus* CSN2 alleles described in the literature [3,5–7].

Nucleotide Position *	SNP Name **	Allele (Nucleotide/Amino Acid)									
		A1	A2	A3	B	C	I	D	E	F	G
6690	CSN2_2	G/Glu	G/Glu	G/Glu	G/Glu	A/Lys	G/Glu				
8101	CSN2_3	A/His	C/Pro	C/Pro	A/His	A/His	C/Pro	C/Pro	C/Pro		
8178	CSN2_4	A/Met	A/Met	A/Met	A/Met	A/Met	C/Leu				
8219	CSN2_5	C/His	C/His	A/Gln	C/His	C/His	C/His				
8267	CSN2_6	C/Ser	C/Ser	C/Ser	G/Arg	C/Ser	C/Ser				Leu
8356	CSN2_1	C/Prp								T/Leu	

*on the basis of the bovine genomic sequence GenBank Accession No. X14711; ** the name assigned to SNP that was present in microarrays.

Table S4. Selected CSN3 genetic variants in relation to nucleotide position, amino acid change within various *Bos genus* CSN3 alleles described in the literature [3,5–8].

Nucleotide Position *	SNP Name **	Allele (Nucleotide/Amino Acid)								
		A	A1	B	C	D	E	G1	H	I
12950	CSN3_1	C/Arg						T/Cys		
12951	CSN3_2	G/Arg		G/Arg	A/His	A/His	G/Arg		G/Arg	
12971	CSN3_3	T/Ser								G/Ala
13065	CSN3_4	C/Thr		C/Thr	C/Thr		C/Thr		T/Ile	
13068	CSN3_5	C/Thr		T/Ile	T/Ile					
13104	CSN3_6	A/Asp		C/Ala	A/Asp		A/Asp		A/Asp	
13111	CSN3_7	A/Pro	G/Pro							
13124	CSN3_8	A/Ser		A/Ser	A/Ser		G/Gly		A/Ser	
13165	CSN3_9	A/Ala		G/Ala	A/Ala		A/Ala		A/Ala	

*on the basis of the bovine genomic sequence GenBank Accession No. AY380228; ** the name assigned to SNP that was present in microarrays.

Table S5. Selected PAEP genetic variants in relation to nucleotide position, amino acid change within various *Bos genus* PAEP alleles described in the literature [3,5–7].

Nucleotide Position *	SNP Name **	Allele (Nucleotide/Amino Acid)		
		A	B	D
3065	PAEP_1		G/Glu	C/Gln
3982	PAEP_2	T/Asp	C/Gly	
5174	PAEP_3	C/Asn	T/Asn	
5223		G/Val	T/Val	
5261		GGT/Val	GGC(TGC)/Ala	
5263	PAEP_4	T/Val	C/Ala	

*on the basis of the bovine genomic sequence GenBank Accession No. X14710; ** the name assigned to SNP that was present in microarrays.
